# BENEFICIAL EFFECTS OF CONTACT WITH OLDER ADULTS FOR AGE STEREOTYPES AND SELF-VIEWS OF AGING

**DOI:** 10.1093/geroni/igac059.505

**Published:** 2022-12-20

**Authors:** Thomas Hess, Sylvie Graf, Shyhnan Liou, Clara Paulo de Couto, Helene Fung, Jana Nikitin, Klaus Rothermund, Ella Cohn-Schwartz

**Affiliations:** North Carolina State University, Raleigh, North Carolina, United States; Czech Academy of Sciences, Brno, Moravskoslezsky kraj, Czech Republic; National Cheng Kung University, Tainan, Tainan, Taiwan (Republic of China); Friedrich Schiller University Jena, Jena, Thuringen, Germany; The Chinese University of Hong Kong, Hong Kong, Hong Kong; University of Vienna, Vienna, Wien, Austria; Friedrich Schiller University Jena, Jena, Thuringen, Germany; Ben-Gurion University, Beer-Sheva, HaDarom, Israel

## Abstract

Intergenerational contact is beneficial for improving attitudes toward older people, including age stereotypes (AS). To date, however, research on the topic has focused on younger adults (intergenerational contact), overlooking the possible perks for older adults themselves (contact with same-age peers). The current study investigated the association between contact with older adults and views of the self in old age (VSOA) among younger and older adults in a domain-specific way. The sample comprised younger (39-55 years, n = 1,012) and older (65-90 years, n = 1,344) adults from the Ageing as Future international study. Findings indicated that contact with older adults was related to more positive VSOA and this was partly mediated by AS. These relations were stronger for older adults, indicating that interactions with other older adults may help favorably shape how older adults view their ingroup and aging. Beneficial effects emerged mostly in the friends and leisure domains.

